# Identification of Novel Tumor Microenvironment Regulating Factor That Facilitates Tumor Immune Infiltration in Cervical Cancer

**DOI:** 10.3389/fonc.2022.846786

**Published:** 2022-06-29

**Authors:** Jingjing Xu, Zhe Huang, Yishu Wang, Zhenxian Xiang, Bin Xiong

**Affiliations:** ^1^ Department of Gastrointestinal Surgery & Department of Gastric and Colorectal Surgical Oncology, Zhongnan Hospital of Wuhan University, Wuhan, China; ^2^ Hubei Key Laboratory of Tumor Biological Behaviors, Wuhan, China; ^3^ Hubei Cancer Clinical Study Center, Wuhan, China; ^4^ Department of Electrical and Computer Engineering, University of Illinois at Urbana-Champaign, Urbana, IL, United States; ^5^ Department of Legal English and TOEIC, Adelaide University, North Terrace, SA, Australia

**Keywords:** CXCR3, tumor microenvironment, estimate, CIBERSORT, PD-1, cervical cancer

## Abstract

Cervical cancer is one of the most common gynecologic malignancies and one of the leading causes of cancer-related deaths in women worldwide. There are more than 30 categories of human papillomavirus infections in the genital tract. The recently discovered immune checkpoint suppression is a potential approach to improve clinical outcomes in these patients by altering immune cell function. However, many questions remain unanswered in terms of this method. For example, the proportion of responders is limited and the exact mechanism of action is uncertain. The tumor microenvironment (TME) has long been regarded as having nonnegligible influence on effectiveness of immunotherapy. The programmed cell death protein 1 (PD-1) pathway has received much attention due to its involvement in activating T-cell immune checkpoint responses. Since tumor cells may evade immune detection and become highly resistant to conventional treatments, anti-PD-1/PD-L1 antibodies are preferred as a kind of cancer treatment and many have just been licensed. To provide a theoretical basis for the development of new therapies, investigating the effect of tumor microenvironment on the prognosis of cervical cancer is necessary. In this work, immunological scores obtained from the ESTIMATE algorithm were used to differentiate between patients with high and low immune cell infiltration. We identified 11 immunologically significant differentially expressed genes (DEGs). For example, CXCR3 was found to be an important factor in CD8^+^ T cell recruitment and tumor immunological infiltration in cervical cancer. These results may lead to novel directions of understanding complex interactions between cancer cells and the tumor microenvironment, as well as new treatment options for cervical cancer.

## Introduction

Cervical cancer is a type of gynecological cancer very harmful to women’s health (1, 2). Human papillomavirus (HPV) infection, smoking, and a compromised immune system are all risk factors for cervical cancer (3, 4). Because persistent HPV infection is associated with immune system dysfunction and the development of cervical cancer (5), cervical cancer is a promising target audience for treatment through the use of immunotherapeutic means (6). Various immunomodulatory therapies, such as bacterial vaccine vectors and T-cell therapies, have been investigated. In the case of recurrence or metastasis, immune checkpoint inhibition in combination with chemoradiotherapy for definitive treatment shows potential (7, 8). Therefore, it is crucial to investigate the oncogenic process and treatment of cervical cancer.

The significance of the tumor microenvironment (TME) in tumor development is evidenced by an increasing number of studies (6). Collaboration between cancer cells and their supporting cells, such as immortal proliferation, resistance to apoptosis, and evasion of immune surveillance, influences the malignant phenotype of cancer (9). Thus, TME has a significant impact on the therapeutic response and clinical prognosis of cancer patients (10, 11). Cervical cancer is a HPV-related cancer, and the tumor microenvironment (TME) plays an important role in its progression. Tumor epithelial cells and other tumor-supporting cells such as immune cells, fibroblasts, immunosuppressive cells, adipocytes, endothelial cells, and pericytes constitute the tumor microenvironment, which is a complex and dynamic biome (12). To promote tumor angiogenesis, proliferation, invasion, and metastasis, and to dictate treatment-resistant activities, cancer cells rapidly attract supporting cells from the surrounding endogenous host stroma (13). Tumor-infiltrating immune cells (TICs) are promising prognostic indicators because they are closely associated with tumor growth (14). TICs are crucial in cervical cancer prognosis and are associated with overall survival (15). The CXCL10/CXCR3 signaling pathway regulates leukocyte trafficking and angiogenesis through paracrine interactions between tumor and stromal cells (16). Through the JAK and STAT signaling pathways, CXCL10-CXCR3 is hypothesized to regulate PD-L1 synthesis in fibroblasts (17). Activation of PD-L1/PD-1 poses great difficulties for cancer therapy (18), which is often associated with cytotoxic T lymphocyte malfunction. In patients with gynecologic malignancies, elevated PD-L1 expression may be a useful biomarker for predicting clinical outcomes (19, 20). Based on these findings, adaptive immunity in the tumor microenvironment is crucial for cervical cancer treatment. Therefore, it is difficult to find precise genetic tests to determine the dynamic regulation of immune and stromal components in the TME.

How different cell types affect regulation of TME was highlighted by transcriptome sequencing patterns and functional genomics analysis. The number of TICs and the proportion of immune and stromal components in CESC samples from The Cancer Genome Atlas (TCGA) database were calculated using ESTIMATE and CIBERSORT algorithms in the R software. A predictive biomarker, CXC chemokine receptor-3 (CXCR3), was identified. The chemokine receptor CXCR3 has three different ligands. Three alternative CXCLs exist, which are CXCL9, CXCL10, and CXCL11 (21). In the tumor microenvironment, CXCL10 has been revealed to have anti-malignant properties. According to the recent study, elevated PD-L1 expression *via* the CXCL10-CXCR3 axis improves viral latency and immune evasion of fibroblasts (17). Therefore, CXCR3 may participate in CESC TME and immunotherapy. By examining differentially expressed genes (DEGs) generated by immune and stromal components in CESC samples, we found that CXCR3 may be a viable biomarker for TME alteration and immunotherapeutic status in CESC. The tumor microenvironment is largely thought of as immunosuppressive, leading to CD8^+^ T lymphocyte dysfunction and thus promoting tumor development. The presence of high concentrations of CD8^+^ T cells in tumor tissue is a positive prognostic indicator in many cancers. Blockade of the suppressive programmed cell death 1 (PD-1) pathway produces a therapeutic response in a variety of tumor types (22). At the same time, the majority of patients do not respond or have disease recurrence, necessitating additional studies (23). Recent studies found the CXCR3 chemokine system is a biomarker of PD-1 blockade sensitivity, and increasing the intratumoral activity of this chemokine system may improve treatment efficacy (24). However, only a proportion of patients respond to PD-1 immune checkpoint blockade, emphasizing the need for a better understanding of the underlying mechanisms of PD-1-inhibitor-mediated enhancement of the anti-tumor CD8^+^ T cell response (25). Finding new ways to understand how cancer interacts with the tumor microenvironment can lead to discoveries which can be used to generate prognostic assessments and clinical treatment options.

## Materials and Methods

### Raw Data

The TCGA database was accessed to obtain transcriptomic RNA-seq data and clinical data from 309 CESC patients (normal samples, 3 cases; tumor samples, 306 cases). (https://portal.gdc.cancer.gov/).

### Generation of ImmuneScore, StromalScore, and ESTIMATEScore

The ESTIMATE algorithm uses the estimation package installed in R language version 4.1.0 to estimate the immune and stromal components of the TME for each sample, expressed as three scores. The total proportion of immune, stromal and both components of the TME was positively correlated with Immunocore, StromalScore, and ESTIMATEScore, which means the higher the correlation value, the higher the proportion of the corresponding component of the TME.

### Survival Analysis

Analyses were performed using the survival and survminer packages loaded with the R language, as well as the Kaplan-Meier plotter website (https://kmplot.com/analysis/index.php?p=service&cancer=pancancer_rnaseq). For survival analysis, exact survival ranging from 0 to 17.6 years was recorded for all tumor samples. Statistical significance was determined using the log-rank test, and p<0.05 was considered significant. To create survival curves, the Kaplan-Meier method was utilized, and the log-rank test was performed to determine statistical significance.

### Generation of DEGs Between High-Score and Low-Score Groups Regarding ImmuneScore and StromalScore

The dataset of 306 tumor samples were classified as high or low scoring based on comparison with the median immune and stromal scores. Differential gene expression analysis was performed using the limma package, and DEGs were constructed by comparing samples with high and low scores. After logarithmic (high group/low group) transformation, DEGs with a change greater than 1 and false discovery rate (FDR) less than 0.05 were judged to be significant.

### Heatmaps

The pheatmap package in the R language was used to construct heatmaps of stromal and immune DEGs.

### Difference Analysis of Scores With Clinical Stages

The TCGA database was used to collect the clinicopathological characteristics of the CESC samples. The study was carried out in the R language. Depending on the number of clinical stages, the Wilcoxon rank-sum or the Kruskal-Wallis rank-sum test was used to determine significance.

### GO and KEGG Enrichment Analysis and PPI Network Construction of DEGs

GO and KEGG enrichment analysis was performed on 425 DEGs using R language with packages clusterProfiler, enrichplot, and ggplot2. Results were considered significantly enriched at a significance threshold p of 0.05. The STRING database (https://string-db.org/) was used for online PPI network analysis. These protein interaction data were imported using Cytoscape version 3.8.0. Nodes with an interaction confidence level greater than 0.95 were used to build the network.

### COX Regression Analysis

Survival package for univariate COX regression are available in R. Univariate Cox regression analysis was conducted to explore the impact of each gene on overall survival. The top 114 genes for univariate COX are displayed in the graph, arranged by p-value from the lowest to the highest.

### Gene Set Enrichment Analysis

Gene Set Enrichment Analysis (GSEA) is a method for interpreting biological meaning of a list of genes by the analysis of the overlaps with various previously defined gene sets (26). Kegg.v7.3.symbols.gmt were downloaded from Molecular Signatures Database as the target sets using the software GSEA 4.0.3 downloaded from Broad Institute. Only the gene sets with NOM p<0.05 were considered to be significant after the whole transcriptome of all tumor samples were applied by GSEA.

### TICs Profile

Using the CIBERSORT, a method for characterizing the abundances of member cell types in a mixed cell population from their gene expression profiles (27), the TIC abundance profiles of all tumor samples were analyzed, and only 250 tumor samples with a p<0.05 were chosen for further investigation using quality filtering.

## Results

### Analysis Process of This Study

**Figure 1:**
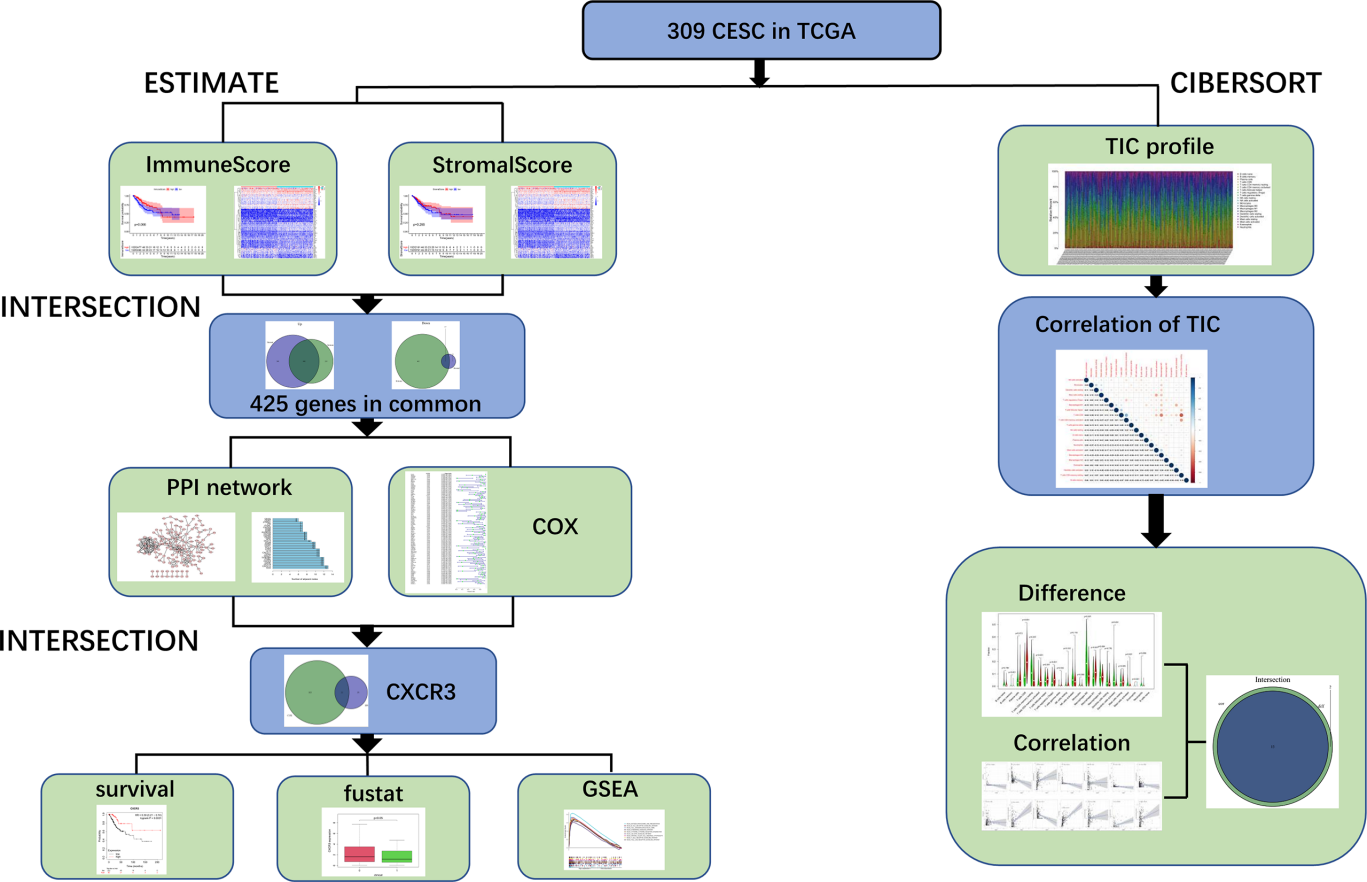
Analysis workflow of this study.

**Figure 1** depicts the analytical approach in our study. To assess the proportion of TICs and the number of immune and stromal components in the CESC samples, we extracted transcriptomic RNA-seq data from the TCGA database for 309 patients using the CIBERSORT and ESTIMATE techniques. The ESTIMATE algorithm is a method for estimating the proportion of TICs and the amount of immune and stromal component in CESC samples. A PPI network and univariate COX regression analysis were created using DEGs shared by ImmuneScore and StromalScore, and intersection analysis were performed using the core nodes of the PPI network and the top prognostic genes obtained from the univariate COX regression analysis. After obtaining 11 genes, we conducted a series of studies on CXCR3, including survival and COX regression, Gene Set enrichment analysis (GSEA), as well as association with TICs.

### Scores Were Correlated With the Survival of CESC Patients

**Figure 2:**
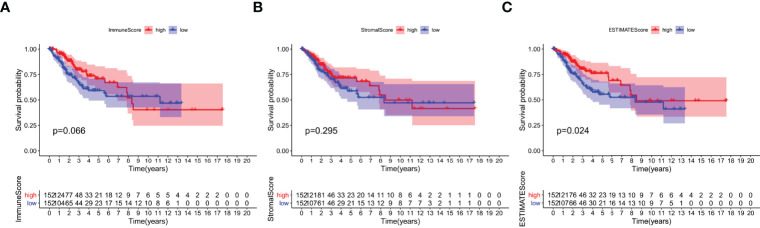
Correlation of scores with the survival of patients with CESC. **(A)** Kaplan–Meier survival analysis for CESC patients grouped into high or low score in ImmuneScore determined by the comparison with the median. p = 0.066 by log-rank test. **(B)** Kaplan–Meier survival curve for StromalScore with p = 0.0295 by log-rank test. **(C)** Survival analysis with Kaplan–Meier method for CESC patients grouped by ESTIMATEScore (p = 0.024 by log-rank test).

To study the correlation between immune and stromal component ratios and the survival rate, survival analysis was used for ImmuneScore, StromalScore, and ESTIMATEScore respectively. The estimated higher score in ImmuneScore or StromalScore represents the larger amount of the immune or stromal component ratios in TME. ESTIMATEScore is the sum of ImmuneScore and StromalScore denoting the comprehensive component ratio of both of them in TME. As shown in **Figures 2A, B**, ESTIMATEScore and StromalScore had no significant correlation with the overall survival rate. ESTIMATEScore still showed positive correlation with the survival rate (**Figure 2C**). These results implied that the immune and stromal components in TME were significant for indicating the prognosis of CESC patients.

### Scores Were Associated With the Clinicopathological Features of CESC Patients

**Figure 3:**
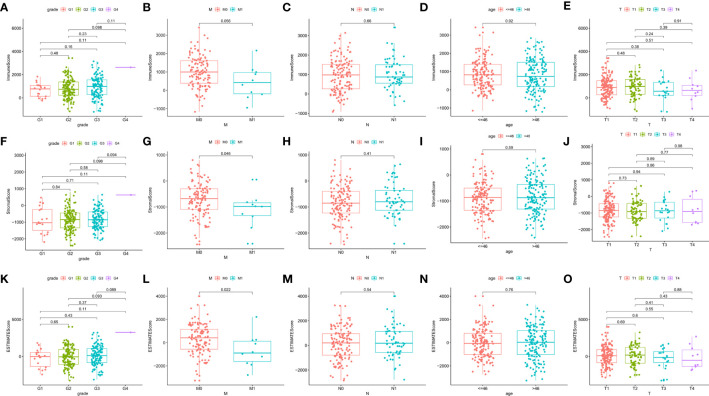
Correlation of ImmuneScore and StromalScore with clinicopathological staging characteristics. **(A–E)** Distribution of ImmuneScore in grade, M, N, age, and T classification. The p value is calculated by Kruskal–Wallis rank sum test. **(F–J)** Distribution of StromalScore in grade, M, N, age, and T classification. The p value is calculated by Kruskal–Wallis rank sum test. **(K–O)** Distribution of ESTIMATEScore in grade, M, N, age, and T classification. The p value is calculated by Kruskal–Wallis rank sum test.

Clinical data from CESC patients in the TCGA database were analyzed to study the correlation between immune and stromal component ratios and clinicopathological features. We analyzed the correlation of grade, TNM stages M classification, N classification, age, as well as T classification with ImmuneScore (**Figures 3A–E**), StromalScore (**Figure 3F–J**) and ESTIMATEScore (**Figures 3K–O**). StromalScore and ESTIMATEScore were found to be negatively correlated with M classification of TNM stages (**Figure 3G**, p=0.046 and **Figure 3L**, p=0.022, respectively). The ratio of immunological and stromal components was related to the development of CESC, such as invasion and metastasis, as shown in **Figures 3G, L** (p=0.046 and p=0.022, respectively).

### Identification and Functional Analysis of DEGs

**Figure 4:**
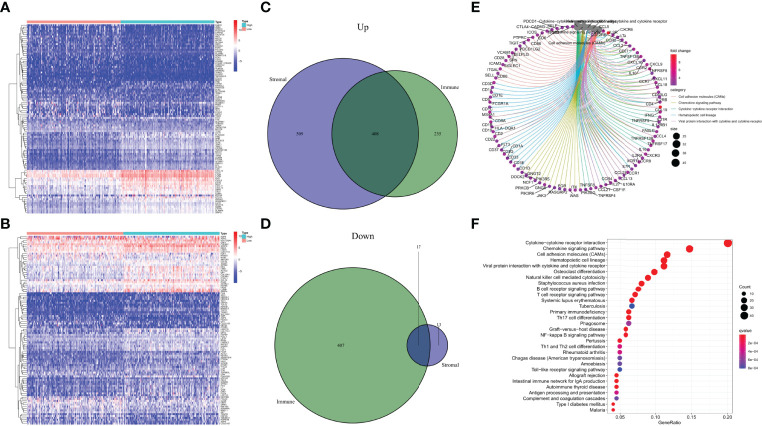
Heatmaps, Venn plots, and enrichment analysis of GO and KEGG for DEGs. **(A)** Heatmap for DEGs generated by comparison of the high score group vs. the low score group in ImmuneScore. Row name of heatmap is the gene name, and column name is the ID of samples which not shown in plot. Differentially expressed genes were determined by Wilcoxon rank sum test with q = 0.05 and fold-change>1 after log2transformation as the significance threshold. **(B)** Heatmap for DEGs in StromalScore, similar with **(A)**. **(C, D)** Venn plots showing common up-regulated and down-regulated DEGs shared by ImmuneScore and StromalScore, p < 0.05 and fold-change>1 after log_2_ transformation as the DEGs significance filtering threshold. **(E, F)** GO and KEGG enrichment analysis for 425 DEGs, terms with p < 0.05 were believed to be enriched significantly.

The specific alterations of gene profiles associated with immunological and stromal components in TME were discovered by a comparative investigation of high-scoring and low-scoring samples. In contrast to the median, ImmuneScore (high vs. low scoring samples) yielded a total of 1067 DEGs, with 643 genes upregulated and 424 genes downregulated (**Figures 4A–D**). Similarly, StromalScore identified 947 genes, of which 917 were up-regulated and 30 were down-regulated (**Figures 4A–D**). The Venn diagram’s intersection analysis shows that 408 up-regulated genes had higher ImmuneScore and higher StromalScore, whereas 17 down-regulated genes had lower scores. These DEGs may be used to assess the status (425 genes in total). These results closely matched immune-related GO terms, such as the interaction of hematopoietic cell lines and viral proteins with cytokines and cytokine receptors, according to gene ontology (GO) enrichment analyses (**Figure 4E**). Natural killer cell-mediated cytotoxicity, S. aureus infection, B-cell receptor signaling pathways, and T-cell receptor signaling pathways were also discovered by KEGG enrichment analysis (**Figure 4F**). As a result, overall function of DEGs appears to engage in immune-related activities, showing that immunological factors are a key aspect of TME in CESC.

### Intersection Analysis of PPI Network and Univariate COX Regression

**Figure 5:**
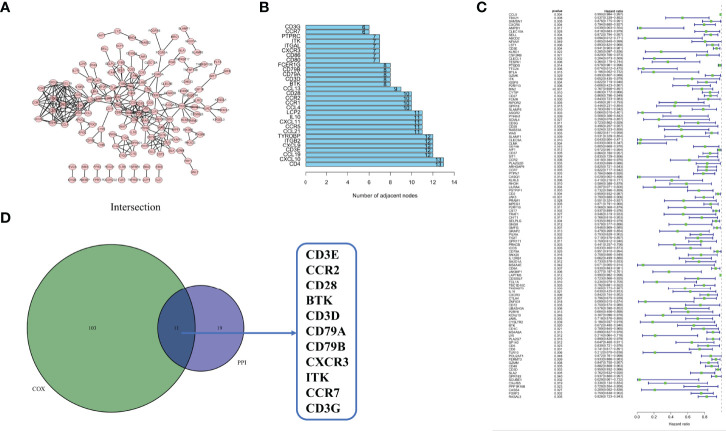
Protein–protein interaction network and univariate COX. **(A)** Interaction network constructed with the nodes with interaction confidence value > 0.95. **(B)** The top 30 genes ordered by the number of nodes. **(C)** Univariate COX regression analysis with 425 DEGs, listing the top significant factors with p < 0.05. **(D)** Venn plot showing the common factors shared by leading 30 nodes in PPI and top significant factors in univariate COX.

To examine the underlying mechanisms of the correlation of the genes, we used Cytoscape software to establish a PPI network based on the STRING database. As shown in **Figure 5A**, 425 genes interacted with each other, and the top 30 genes, ranked by several nodes, are represented as a histogram (**Figure 5B**). We used univariate COX regression analysis on the survival of CESC patients to find the top prognostic genes among the 425 DEGs (**Figure 5C**). Only 11 genes, CD3E, CCR2, CD28, BTK, CD3D, CD79A, CD79B, CXCR3, ITK, CCR7, and CD3G, were identified to overlap in a intersection analysis between the leading nodes in the PPI network and the top 104 genes ordered by p-value in the univariate COX model (**Figure 5D**).

### The Correlation of CXCR3 Expression With the Survival in CESC Patients and the Potential to Be an Indicator of TME Modulation

**Figure 6:**
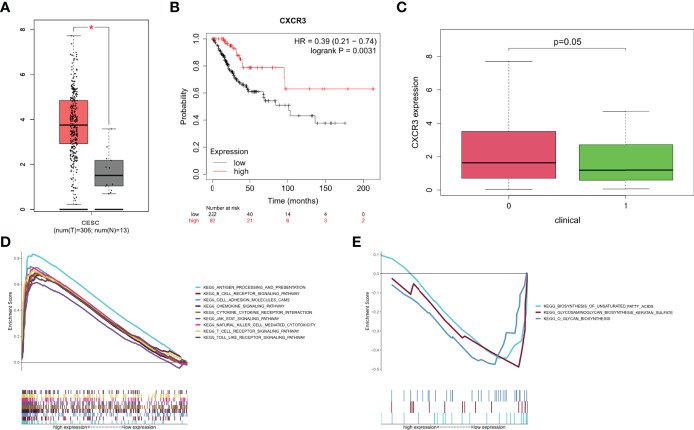
Survival analysis, clinical characteristics and GSEA for samples with high CXCR3 expression and low expression of CESC patients. **(A)** Differentiated expression of CXCR3 in the normal and tumor sample. **(B)** Survival analysis for CESC patients with different CXCR3 expression. Patients were labeled with high expression or low expression. **(C)** The correlation of CXCR3 expression with survival stage(fustat: 0 = survival, 1 = death). **(D)** Enriched gene sets in KEGG of high CXCR3 expression. Only several leading gene sets are shown in plot. **(E)** Enriched gene sets in KEGG by the low CXCR3 expression.

The tumor microenvironment is immunosuppressive, and contributes to tumor growth by causing the malfunction of CD8^+^ T cells. According to CXCR3 analysis, CXCR3 expression was significantly higher in tumor samples than in normal samples (**Figure 6A**). Based on the median CXCR3 expression, all CESC samples were divided into two groups, high expression of CXCR3 and low expression of CXCR3. According to our study, CESC patients with high CXCR3 expression lived longer than those with low CXCR3 expression (**Figure 6B**). The final clinical characterization study yielded similar results, with greater CXCR3 expression in patients who were alive (**Figure 6C**). According to the results of the study, CXCR3 expression in TME was associated with a better prognosis in CESC patients. This may be due to the anti-tumor response of CXCR3-signaling CD8^+^ T cells in the tumor microenvironment, which leads to a better prognosis of patients (23). This may explain why CXCR3 expression is higher in cervical cancer, but it may also indicate a better prognosis for patients with cervical cancer. Since the level of CXCR3 was favorably associated with the survival of CESC patients, GSEA was used to respectively compare the samples of CXCR3 expression in the high and low expression groups. As shown in **Figure 6D**, the CXCR3 high presentation group was enriched in antigen processing and presentation, B-cell receptor signaling, chemokine signaling, natural killer cell-mediated cytotoxicity, T-cell receptor signaling, and toll-like receptor signaling. In the CXCR3 low expression group, genes related to metabolic pathways such as biosynthesis of unsaturated fatty acids, glycosaminoglycan biosynthesis, keratin sulfate, and O-glycan biosynthesis were identified (**Figure 6E**). These results suggest that CXCR3 may be used as an indicator of TME status.

### Correlation of CXR3 With the Proportion of TICs

**Figure 7:**
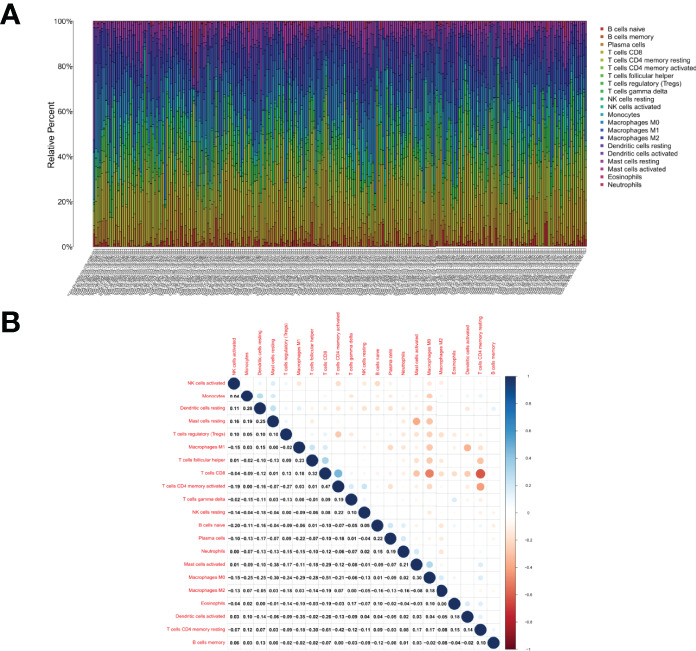
TIC profile in tumor samples and correlation analysis. **(A)** Barplot showing the proportion of 21 kinds of TICs in CESC tumor samples. Column names of plot were sample ID. **(B)** Heatmap showing the correlation between 21 kinds of TICs and numeric in each tiny box indicating the p value of correlation between two kinds of cells. The shade of each tiny color box represented corresponding correlation value between two cells, and Pearson coefficient was used for significance test.

The fraction of tumor-infiltrating immune subpopulations was determined using CIBERSORT, and 21 different types of immune cells in tumor tissues were created to further support the link between CXCR3 expression and the TME (**Figure 7**). As detailed below, the differences and correlations were studied.

**Figure 8:**
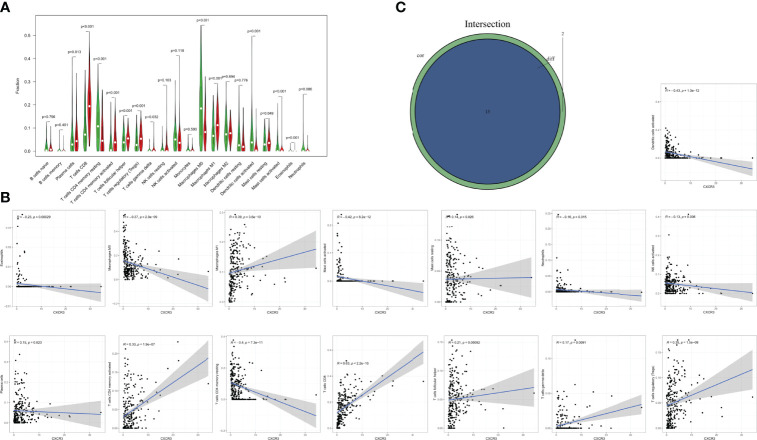
Correlation of TICs proportion with CXCR3 expression. **(A)** Violin plot showed the ratio differentiation of 21 kinds of immune cells between CESC tumor samples with low or high CXCR3 expression relative to the median of CXCR3 expression level, and Wilcoxon rank sum was used for the significance test. **(B)** Scatter plot showed the correlation of 15 kinds of TICs proportion with the CXCR3 expression (p<0.05). The blue line in each plot was fitted linear model indicating the proportion tropism of the immune cell along with CXCR3 expression, and Pearson coefficient was used for the correlation test. **(C)** Venn plot displayed 13 kinds of TICs correlated with CXCR3 expression codetermined by difference and correlation tests displayed in violin and scatter plots, respectively.

According to differential and correlation analyses, CXCR3 expression was linked to a total of 13 TICs (**Figure 8**). M1 macrophages, resting mast cells, plasma cells, activated memory CD4^+^ T cells, CD8^+^ T cells, T follicular helper cells, gamma-delta (γδ) T cells, and regulatory T cells (Tregs) were found to be positively correlated with CXCR3 expression. Five TICs, including activated dendritic cells, eosinophils, M0 macrophages, activated mast cells, and resting memory CD4^+^T cells, were found to be negatively correlated with CXCR3 expression. These findings back up the hypothesis that CXCR3 levels impact TME immune activation.

## Discussion

The purpose of this study is to seek for genes associated with TME that affect survival and TNM staging in CESC patients in the TCGA database. Based on a series of bioinformatic studies, CXCR3 may be an indicator of TME status in CESC patients. The immune system function is associated with CXCR3 (28). Future work can be focused on helping modify the therapeutic purpose of TME and the relationship between TME and tumors (29). The role of the immune microenvironment in cancer has been demonstrated in several studies. According to our transcriptome analysis on the CESC data in the TCGA database, the immune component of TME has an impact on patient prognosis. In particular, the ratio of immune and stromal components in TME was found to be strongly associated with the development of CESC including invasion and metastasis. These findings shed light on the importance of studying tumor-immune cell interactions as they provide new insights into how to develop more effective therapeutic options (30).

It is crucial to have a comprehensive understanding of the complex interplay of receptors-cell death cascades in inflammatory tumor microenvironment in cervical cancer (31). Previous research has shown that immunogenic cell death and cancer stem cells-related innovative clinical and translational research provided patients a boon in targeted immunotherapy in gynecologic malignancies including cervical cancer as well as other inflammatory cancers (32, 33). PD-L1 is an important suppressive immune receptor involved in the immunosuppression of cancer (34). Recent studies have found that PD-L1 is overexpressed in cervical cancer and its knockdown reduces the proliferation, invasiveness, and oncogenicity of cervical cancer cells (35). Three chemokine ligands of CXCR3 are CXCL9, CXCL10, and CXCL11 (21). CXCL10 has been regarded as having an anti-malignant role in the tumor microenvironment (36). According to recent work, the CXCL10-CXCR3 axis enhances viral latency and immune evasion of fibroblasts through upregulation of PD-L1 expression (17). Thus, CXCR3 may be a part of CESC TME and immunotherapy. The CXCR3 chemokine system was found to be a biomarker of PD-1 blockade sensitivity in recent studies, and therapeutic efficacy may be improved by enhancing the intratumoral activity of this chemokine system (24). Therefore, we investigated the association between CXCR3 expression and TME. According to GSEA data, immune-related signaling pathways were significantly enriched in the CXCR3 high expression group, including natural killer cell-mediated cytotoxicity, S. aureus infection, B-cell receptor signaling pathway, and T-cell receptor signaling pathway. The CXCR3 low expression group regarding unsaturated fatty acid production, glycosaminoglycan biosynthesis, keratin sulfate, and O-glycan biosynthesis were enriched. Based on these findings, we argue that CXCR3 may function in the immunity and metabolism of TME.

In this work, a fraction of TICs was analyzed using CIBERSORT, and a good association between CD8^+^ T cells and CXCR3 expression was found in CESC patients. For some malignancies, the presence of a high number of CD8^+^ T lymphocytes in the tumor tissue is a good prognostic signal (37). There are some studies focused on the application of prognostic and therapeutic TILs in cervical cancer. A study compared lymphocytes in cervical tissues from 19 patients with CIN and from 20 patients with normal cervices. The proportion of CD8^+^ T cells is significantly increased in the dysplastic tissue, which clearly indicates that a higher density of CD8^+^ TILs are positively associated with the progress of cervical cancer (38). A systematic review with meta-analysis on the prognostic significance of TILs showed that CD8^+^ lymphocytes had a positive effect on patient overall survival (OS) of cervical cancer with a hazard ratio (HR) of 0.71, indicating that CD8^+^ TILs have prognostic values for not only cancer progression but also patient OS (39). A finding from phase II trials indicates that TILs therapy may prove an efficacious option for cervical cancer. The treatment was among 27 patients with metastatic cervical carcinoma refractory to the standard of care. This response was seen sustainedly in patients whose disease was refractory to standard treatment (40, 41). CXCR3 seems to be important for maintaining the immune activity of TME, as there is a positive correlation between CD8^+^ T cell numbers and CXCR3 expression in CESC patients (42). Based on the increase in CD8^+^ T cells, CXCR3 may have antitumor efficacy in CESC. These molecular analyses at the tissue and cell level could reveal the cellular status of TME, but information related to spatial-level cellular distribution, co-organization, and cell-cell interaction is insufficient in the TME (43). Deep spatial analysis is capable of unraveling tumor evolutionary trajectories as well as geospatial evolution in cell populations and their expression signatures (44). In cervical tumors, spatial analysis data could map the spatial relationships between different cell phenotypes. Spatial analysis is able to distinguish different cell populations that play specific roles in activation and regulation close to the malignant cells, suggesting that those cells can play specific roles according to their distribution. As is reported in lung adenocarcinoma, the close proximity and interaction between malignant cells and T-cells expressing PD-L1 and PD-1 suggest that cytotoxic T-cells actively interface with the malignant cells and may increase the risk of tumor recurrence in those patients (45). Recent study provides functional and spatial analysis of immune cells in the Lymphocyte-rich classic Hodgkin lymphoma (LR-CHL) microenvironment at single-cell resolution. It identifies a unique CD4^+^PD-1^+^CXCL13^+^CXCR5^-^ TFH-like subset that surrounds Hodgkin Reed-Sternberg (HRS) cells, appears in close proximity to CXCR5^+^ B cells, and is associated with poor clinical outcome. This finding suggests the pathogenic mechanism of the CXCL13/CXCR5 axis and PD-1^+^CXCL13^+^ T cells as a treatment target in LR-CHL. This reveals that the expression profiles of CXC motif cytokines and their receptors have a crucial impact in the TME and patient prognosis at the tumor spatial-level. Several studies showed that checkpoint blockades targeting PD-1/PD-L1 pathways have achieved efficient clinical responses by suppressing cancer progression and improving survival in cervical cancer (46). We can infer that CXCL10/CXCR3 axis and PD-1^+^CXCL10^+^ T cells may also play specific roles corresponding to their distribution in cervical cancer. Therefore, more studies are needed to determine the precision of combining CXCR3 expression, tumor-infiltrating T-cell isoforms, and anti-PD-1/PD-L1 treatment response in patients with cervical cancer. In addition, we need to validate the findings of the study through experiments *in vitro* and *in vivo*. We also should admit that the TCGA data has limitations. The TCGA isoform expression quantification data annotation errors and batch effects may occur due to the fact that the data was collected not only at different times but also by different institutions (47).

## Data Availability Statement

The original contributions presented in the study are included in the article/supplementary materials. Further inquiries can be directed to the corresponding author.

## Ethics Statement

This study was approved by the Zhongnan Hospital of Wuhan University ethics committee.

## Author Contributions

JX designed the research, completed data analysis and completed the manuscript. ZH and YW helped to revise the manuscript. JX, ZX and BX helped with the final correction. All authors approved the final version.

## Conflict of Interest

The authors declare that the research was conducted in the absence of any commercial or financial relationships that could be construed as a potential conflict of interest.

## Publisher’s Note

All claims expressed in this article are solely those of the authors and do not necessarily represent those of their affiliated organizations, or those of the publisher, the editors and the reviewers. Any product that may be evaluated in this article, or claim that may be made by its manufacturer, is not guaranteed or endorsed by the publisher.

## Abbreviations

TME, tumor microenvironment; CESC, cervical squamous cell carcinoma and endocervical adenocarcinoma; PPI, protein–protein interaction; DEG, differentially expressed gene; CXCR3, CXC chemokine receptor-3; GSEA, Gene Set Enrichment Analysis; TIC, tumor-infiltrating immune cell; TIL, tumor-infiltrating lymphocyte.
